# Germline IGHV3-53-encoded RBD-targeting neutralizing antibodies are commonly present in the antibody repertoires of COVID-19 patients

**DOI:** 10.1080/22221751.2021.1925594

**Published:** 2021-06-06

**Authors:** Qihong Yan, Ping He, Xiaohan Huang, Kun Luo, Yudi Zhang, Haisu Yi, Qian Wang, Feng Li, Ruitian Hou, Xiaodi Fan, Pingchao Li, Xinglong Liu, Huan Liang, Yijun Deng, Zhaoming Chen, Yunfei Chen, Xiaoneng Mo, Liqiang Feng, Xiaoli Xiong, Song Li, Jian Han, Linbing Qu, Xuefeng Niu, Ling Chen

**Affiliations:** a Bioland Laboratory, Guangdong Laboratory of Computational Biomedicine, Guangzhou Institutes of Biomedicine and Health, Chinese Academy of Sciences, Guangzhou, People’s Republic of China; b State Key Laboratory of Respiratory Disease, National Clinical Research Center for Respiratory Disease, Guangzhou Institute of Respiratory Health, the First Affiliated Hospital of Guangzhou Medical University, Guangzhou, People’s Republic of China; c Guangzhou Institute of Infectious Disease, Guangzhou Eighth People's Hospital, Guangzhou Medical University, Guangzhou, People’s Republic of China; d Savaid Medical School, University of Chinese Academy of Science, Beijing, People’s Republic of China; e Microbiome Medicine Center, Department of Laboratory Medicine, Zhujiang Hospital, Southern Medical University, Guangzhou, People’s Republic of China; f iRepertoire Inc. , Huntsville, AL, USA; g College of Life Sciences and Chemistry, Hunan University of Technology, Zhuzhou, People’s Republic of China

**Keywords:** SARS-CoV-2, IGHV3-53, shared clonotype, receptor-binding domain, antibody repertoire sequencing

## Abstract

Monoclonal antibodies (mAbs) encoded by IGHV3-53 (VH3-53) targeting the spike receptor-binding domain (RBD) have been isolated from different COVID-19 patients. However, the existence and prevalence of shared VH3-53-encoded antibodies in the antibody repertoires is not clear. Using antibody repertoire sequencing, we found that the usage of VH3-53 increased after SARS-CoV-2 infection. A highly shared VH3-53-J6 clonotype was identified in 9 out of 13 COVID-19 patients. This clonotype was derived from convergent gene rearrangements with few somatic hypermutations and was evolutionary conserved. We synthesized 34 repertoire-deduced novel VH3-53-J6 heavy chains and paired with a common IGKV1-9 light chain to produce recombinant mAbs. Most of these recombinant mAbs (23/34) possess RBD binding and virus-neutralizing activities, and recognize ACE2 binding site via the same molecular interface. Our computational analysis, validated by laboratory experiments, revealed that VH3-53 antibodies targeting RBD are commonly present in COVID-19 patients’ antibody repertoires, indicating many people have germline-like precursor sequences to rapidly generate SARS-CoV-2 neutralizing antibodies. Moreover, antigen-specific mAbs can be digitally obtained through antibody repertoire sequencing and computational analysis.

## Introduction

The worldwide outbreak of coronavirus disease 2019 (COVID-19), caused by a novel coronavirus named severe acute respiratory syndrome coronavirus 2 (SARS-CoV-2), presents a severe health problem. The surface spike (S) glycoprotein of SARS-CoV-2 engages the cellular receptor angiotensin-converting enzyme 2 (ACE2) via the receptor-binding domain (RBD), which is believed to be a critical target to block viral entry [1,2].

Recently, a series of RBD-targeted mAbs were isolated from SARS-CoV-2 exposed individuals [3–15]. Multiple germline genes were used for the generation of the RBD-targeted mAbs, including VH1-2, VH3-9, VH3-30, and VH3-53. Notably, VH3-53 appeared to be a frequently used heavy chain germline gene among cloned RBD-targeted mAbs, and most of the VH3-53-encoded mAbs showed neutralizing activities [4,8,10,11,12,13,14,15,16]. Several VH3-53-encoded mAbs have been well characterized, such as B-38, CC12.1, C105 and CV30, which neutralize SARS-CoV-2 via blocking of the interaction between RBD and cellular receptor ACE2 [5,10,11,17]. In addition, B-38 and CC12.1 were shown to protect against SARS-CoV-2 challenge in mouse model [10,11]. Anti-RBD antibody response correlated well with neutralizing titres [8,18]; thus, VH3-53 antibodies may play an important role in contributing to protection in SARS-CoV-2 infection. However, previous studies were more focused on the functional investigation of VH3-53-encoded mAbs, the VH3-53-encoded antibodies in the total antibody repertoire in COVID-19 patients have not been investigated. More importantly, the existence and prevalence of shared VH3-53 antibodies among COVID-19 patients were unknown. Antibody repertoire sequencing provides the possibility to address these questions, and it has been successfully used to investigate antibody responses induced by HIV-1, Ebola virus (EBOV), and ZIKA virus (ZIKV) [19–21]. Using this technology, we have previously profiled the ZIKV-elicited antibody response and determined the abundance of ZIKV-specific mAbs in total repertoires [19].

In this study, we performed antibody repertoire sequencing on 40 samples from 13 COVID-19 patients. Antibodyomics analysis was used to identify shared VH3-53 antibodies elicited by SARS-CoV-2 infection. The development of these shared VH3-53 antibodies was longitudinally tracked. Based on computational analysis, we synthesized repertoire-deduced sequences to produce recombinant mAbs for functional confirmation. The RBD binding activities and neutralizing capabilities of these recombinant mAbs were evaluated by enzyme-linked immunosorbent assay (ELISA), biolayer interferometry (BLI) affinity test, surrogate virus neutralization test (sVNT) [22], pseudovirus neutralization assay, structural modeling, and antibody competition assay. Our study provides insight at the antibody repertoire level to understand the generation of shared VH3-53 antibodies targeting to RBD in COVID-19 patients.

## Materials and methods

### Human subjects

A total of 13 confirmed COVID-19 patients aged 33 to 81 were enrolled in this study (Table S1). Five blood samples from 5 healthy controls aged 28 to 58 were also included. All patients were hospitalized at Guangzhou 8th People's Hospital, and SARS-CoV-2 infection status was confirmed by RT-PCR. Single (PtC, PtH, and PtY) or longitudinal (PtF, PtG, PtJ, PtK, PtL, PtP, PtQ, PtS, PtW, and PtZ) blood samples were collected during hospitalization and follow-up visits. Four patients were followed for 3 months after discharge.

### Cell lines

HEK293F is a female human embryonic kidney cell line transformed and adapted to grow in suspension (ThermoFisher, USA). HEK293F cells were cultured in CD 293 TGE Medium (ACRO, China) and place the flask on a shaker platform (120–130 rpm) in the 37°C/5% CO_2_ incubator.

### Isolation of PBMCs and RNA

Peripheral blood mononuclear cells (PBMCs) were isolated with Opti-Prep lymphocyte separation solution (Axis Shield Poc As, Norway) by following the manufacturer’s instructions. Total RNA of PBMCs was extracted using TRIzol™ (ThermoFisher, USA) according to the manufacturer’s instruction.

### Library preparation and antibody repertoire sequencing

Dam-PCR using multiplex primers (iRepertoire, Inc., USA) was performed to amplify the heavy- or light- chain sequences as described previously [19]. Briefly, multiplex primers covering the human VH or VL genes (forward primers) and constant region primers (reverse primers) were designed. The forward primers Fi (forward-in) and reverse primers Ri (reverse-in) also included Illumina paired-end sequencing communal primers B and A, respectively (Illumina, USA). After PCR amplification, the first-round PCR products were used as the template for the second round PCR reaction with Illumina sequencing using communal primers B and A. Unique barcodes introduced in the first round by the constant region primers were used to distinguish the samples. After gel purification using a QIAquick® gel extraction kit (Qiagen, Germany), the resulting product was pooled for high-throughput 250-cycle paired-end sequencing on an Illumina HiSeq 2500 sequencer (Novogene, China).

### Expression of recombinant monoclonal antibody

The antibody heavy- and light-chain V genes (VH/VL) were synthesized (Sino Biological, China) and were amplified and cloned into human IgG1 expression vector by using Clone Express II One Step Cloning Kit (Vazyme, China). The equal amounts of heavy- and light-chain plasmids were transfected into HEK293F cells using EZ Cell Transfection Reagent (Life-iLab Biotech, China) with cells grown to a density of 1 × 10^6^ cells per mL. Following transfection, cells were maintained in CD 293 TGE Medium (ACRO, China) and contained 1/10 CD Feed X supplement (ACRO, China) at 37°C in a humidified 5% CO2 incubator rotated at 120 rpm. After 6 days expression, supernatants were harvested and clarified by centrifugation. Subsequent filtered through 0.22-µm filters and incubated with MabSelect (GE Healthcare, USA) at room temperature for 2 h for antibodies affinity purification. After washing, antibodies were eluted from the MabSelect in chromatography columns using 0.1 M Na-Citrate (pH 3.25) and neutralized with 1M Tris-HCl (pH 8.8). Further purified by centrifugal filtration using Amicon Ultrafilter (Merck Millipore, USA) in PBS and concentrated and stored at −80°C.

### ELISA assay to determine antibody binding activity to SARS-CoV-2 RBD

Coating a 96 well flexible assay plate overnight at 4°C with 100 µL per well of SARS-CoV-2 (2019-nCoV) Spike RBD-His Recombinant Protein (Sino biological, China) at 1 µg/mL in PBS. After standard washing, added 200 µL per well of 5% skim milk to block and incubated for 2 hours at 37°C. Washing wells three times with PBS-0.05% Tween 20 (MP), 100 µL semilogarithmic dilutions in PBS of antibodies were added to each well and incubated at 37°C for 2 hours. After washing wells 3 times with PBS-0.05% Tween 20, plates were incubated with 1:5000 dilutions in 5% skim milk of HRP-labelled Goat Anti-Human IgG(H + L) (Beyotime, China) at 37°C for 1 h. Washing wells 6 times with PBS-0.05% Tween 20, added 100 µL per well of TMB/E Solution (Merck Millipore) and left at room temperature for 15 min. Stop the reaction by adding 50 µL 1 M sulphuric acid and read the OD value at 450 nm.

### Surrogate virus neutralization test

Antibody neutralizing activity was determined by a surrogate virus neutralization test as described previously [22] using cPass™ sVNT kit (Genscript, China) according to the manufacturer’s instruction. Briefly, in separate tubes, mix diluted Positive Control, Negative Control, and the semilogarithmic diluted antibodies with the diluted HRP-RBD with a volume ratio of 1:1 in tubes, incubate the mixtures at 37°C for 30 min. Add 100 µL of the Positive Control mixture, Negative Control mixture, and the semilogarithmic diluted antibodies mixture to the corresponding wells and incubate at 37°C for 15 min. Wash the plate with 260 µL 1× Wash Solution per well for four times. Add 100 µL of TMB Solution to each well and incubate the plate in dark at room temperature for 15 min. Add 50 µL of Stop Solution to each well to stop the reaction, and read the absorbance in the microtiter plate reader at 450 nm immediately.

### Neutralization assay based on pseudotyped lentivirus

Briefly, lentiviral vector pseudotyped by SARS-CoV-2 S protein were produced by co-transfecting the plasmid expressing S protein, which backbone carries an expression cassette for firefly luciferase. Then package this plasmid into 293 T cells to produce pseudotyped lentivirus as previously described [23]. The three times serially diluted antibodies were incubated with the SARS-CoV-2 pseudotyped virus at 37°C for 1 h. The mixture was subsequently incubated with 293T-ACE2 cells for 72 h. The cells were washed twice with PBS and lysed with lysis buffer for luciferase activity. The neutralization titres were calculated as antibodies dilutions at which the luciferase activity was reduced to 50% of that from the virus-only wells.

### Antibodies binding kinetics and competitive binding assay measured by Biolayer interferometry (BLI)

The affinity between SARS-CoV-2 RBD and antibodies were measured by BLI using GatorTM Label-Free Bioanalysis System (GatorBio). All experiments were performed at 25°C with shaking at 1,000 r.p.m, and anti-human IgG Fc biosensors were pre-equilibrated in Q buffer containing PBS (10mM pH7.4), 0.02%Tween-20, and 0.2%BSA for at least 300 s before use in experiments. 10 μg/mL antibody was loaded onto anti-human IgG Fc biosensors for 120 s and flowed the difference in concentrations of SARS-CoV-2 RBD-His recombinant protein (Sino biological) or buffer as control) for 120 s. Then the both dissociate in Q buffer for 300s. The competitive binding between two antibodies was also measured using the GatorTM Label-Free Bioanalysis System (GatorBio). Anti-His biosensors were pre-equilibrated in Q buffer, then 2 μg/mL SARS-CoV-2 RBD-His recombinant protein (Sino biological, China) was loaded onto anti-His biosensors for 120s, and flowed 10 μg/mL of the first antibody for 300 s and 10 µg/mL of the second antibody (or buffer as control) for 300 s. We analysed the data by using Gator™ System Analysis.

### Sequencing data preprocessing and germline gene assignment

The raw FASTQ files were filtered according to their base qualities. 3′ ends of read sequences with quality scores lower than 20 were trimmed using Trimmomatic v0.36 [24]. Following filtering, the paired-end reads were separated according to the unique barcodes at the 5′ end of the reads. The separated paired-end reads were assembled into whole-length antibody sequences based on the overlapping regions using FLASH v1.2.11 [25], requiring a minimum overlapping length of 30 nucleotides. After assembling, the sequences with 300–470 nucleotides were annotated for V(D)J germline genes using MIXCR v3.0.3 [26], using reference V(D)J sequences downloaded from IMGT database (http://www.imgt.org/). Productive sequences that had not stop codons or out-of-frame IGHJ were retained for further analysis.

### Clonal analysis

Productive sequences were assigned to antibody clones by grouping sequences according to their V(D)J germline gene usage using MIXCR v3.0.3 [26]. We defined antibody clones as a group of sequences with the same V gene, the same J gene, and identical CDR3 amino acid sequence. Thus, the total reads of each unique antibody clone were defined as the clone size.

### *De novo* identification of shared VH3-53-encoded clonotypes

VH3-53-encoded heavy chain sequences were extracted from the total repertoires of each individual and then they were pooled together. Single-linkage clustering was performed by comparing the heavy chain complementarity-determining region (HCDR3) amino acid sequences of each antibody clone to identify shared clonotypes. Shared clonotype refers to antibody clones (see section “Clonal analysis”) that derived from different donors but involving the same V gene, the same J gene, the same HCDR3 length, and HCDR3 amino acid sequences of high identity between donors [21]. Here, we clustered all the VH3-53-encoded clones from 13 COVID-19 donors using 80% HCDR3 identity as a threshold. Single-linkage clustering was used in the R package cluster (https://cran.r-project.org/web/packages/cluster/) and the visualization was used the R package igraph (https://cran.r-project.org/web/packages/igraph/index.html). Shared VH3-53-encoded clonotypes presented in this study were further compared with the reported VH3-53-encoded RBD-targeted antibodies.

### Identification of shared light-chain clonotypes based on known VH3-53-encoded RBD-targeted antibodies

A list of VH3-53-encoded RBD-targeted antibodies was compiled from the recently published literature studies [3–15]. VK1-9 repertoires were extracted from the total light-chain repertoires and further compared to the known light chains, respectively. Sequence identities based on overall sequence identity into light-chain variable region were calculated using the R package stringdist v0.9.5.5 (https://cran.r-project.org/web/packages/stringdist/index.html) and normalized to the length of the overall sequence. Within each repertoire, only sequences with a matching V-gene assignment and light-chain complementarity-determining region (LCDR3) length to each RBD-targeted antibody were considered for identity calculating. Here, we selected a threshold of 100% LCDR3 identity and 95% of overall sequence identity for shared clonotype analysis because of the short LCDR3s.

### Phylogenetic analysis

For phylogenetic analysis, the VH3-53 antibody sequences derived from different donors were aligned with each other using MUSCLE v3.8.31 [27] to generate multiple sequence alignment (MSA). Then the MSA was inputted into MEGAX v10.1 [28] to construct a phylogenetic tree using maximal likelihood method with the HKY85 substitution model and bootstrapping (500 replicates). Subsequently, the constructed phylogenetic tree and corresponding MSA were graphically displayed with the R package ggtree v2.2.1 [29].

### Antibody structure modelling and docking

The homology models of VH3-53 antibodies were built on the SWISS-MODEL server (https://swissmodel.expasy.org/) [30]. The crystal structure of SARS-CoV-2 RBD (PDB: 6LZG) were download from Protein Data Bank website (https://www.rcsb.org/), which was determined recently [1]. Docking of the VH3-53 antibodies with SARS-CoV-2 RBD was carried out using the Z-DOCK server (http://zdock.umassmed.edu/) [31], and top 10 potential complexes were considered for further analysis. The most optimal complexes were further prepared by taking the complex structure of B-38/RBD, CV30/RBD or CC12.3/RBD as template. All structural representations were generated using PyMol Molecular Graphics System (https://pymol.org/2/).

### Statistics

Statistical analysis was carried out using GraphPad Prism 8.0 software. Student’s t-test (unpaired, two-tailed) used for the comparison of VH3-53 germline gene usage in heavy chain repertoires between different time points. A *p*-value of less than .05 was considered statistically significant.

## Results

### Germline VH3-53 encoded antibodies increase rapidly after SARS-CoV-2 infection

**Figure 1.**
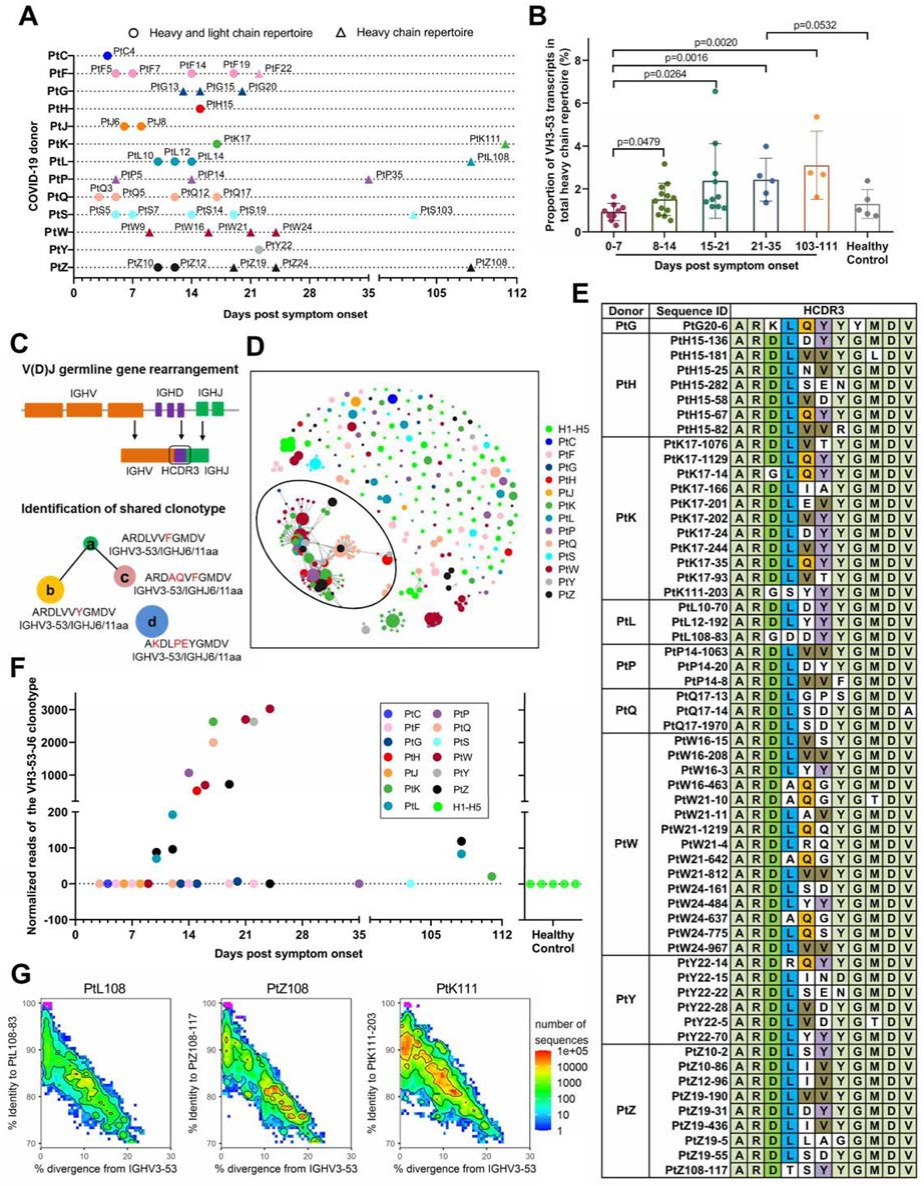
Shared VH3-53-derived antibodies were observed in individuals exposed to SARS-CoV-2. (A) Scheme of longitudinal sample collection and antibody repertoire sequencing. Circles indicate samples used for both heavy- and light-chain repertoire sequencing, while triangles indicate samples used for only heavy-chain repertoire sequencing. (B) Comparison of VH3-53 germline gene usage in heavy chain repertoires between different time points after the onset of symptoms. Statistical tests were performed by Student’s t-test (Unpaired, two-tailed). (C) Scheme of V(D)J gene rearrangement and identification of shared clonotype. Shared clonotype are defined as a group of sequences with the same V/J combination event, the same heavy chain complementary determining region (HCDR3) length, and HCDR3 amino acid sequences of 80% identity between different people. (D) Lineage structure of the VH3-53-J6 clonotype. Each dot represents a unique antibody clone, two dots are connected via a line if they have 80% amino acid similarity on HCDR3 region, the dot area represents the read count for each unique antibody clone, and different colours were used to distinguish different COVID-19 donors and healthy controls. (E) The HCDR3 sequence comparison of the VH3-53-J6 clonotype in the repertoires of different COVID-19 donors. (F) Presence of the shared VH3-53-J6 clonotype in repertoires of COVID-19 patients and healthy people. (G) Identity-divergence plot of three VH3-53 antibody clones (PtL108-83, PtZ108-117, and PtK111-203) from samples collected three months after hospital discharge. All VH3-53 sequences in the repertoires were plotted as a function of sequence divergence from VH3-53 germline gene and sequence identity to PtL108-83, PtZ108-117, and PtK111-203, respectively. Colour gradient indicated sequence density. The sequence with an identical HCDR3 with PtL108-83, PtZ108-117, and PtK111-203 are shown as magenta dots.

We collected 40 blood samples from 13 COVID-19 patients and performed antibody repertoire sequencing (Figure 1(A), Table S1). Samples were collected between day 3 after syndrome onset and up to three months after hospital discharge. Blood samples from five healthy volunteers were used for comparison. SARS-CoV-2 specific IgM and IgG responses were detectable in these COVID-19 patients [32]. The heavy-chain repertoires contain an average of 3.03 ± 1.26 million filtered sequence reads and the light-chain repertoires contain an average of 2.16 ± 0.78 million filtered sequence reads. The total filtered antibody sequences were 196.96 million (Table S1). Germline gene assignment analysis revealed that the median usage of VH3-53 germline gene was 1.55% (ranging from 0.31% to 6.55%) in SARS-CoV-2 exposed individuals, while the usage of VH3-53 germline gene was 1.02% (ranging from 0.75% to 2.38%) in healthy individuals (Table S1). The usage of VH3-53 germline gene increased during the second week after symptom onset. The usage of VH3-53 germline gene maintained at a relatively high level for at least 3 months, indicating the clonal expansion of B cells expressing VH3-53-encoded antibodies after SARS-CoV-2 infection (Figure 1(B)).

We performed a clonal assignment to identify VH3-53-derived public clonotype shared among COVID-19 patients. The public clonotype was defined as a group of sequences with the same V/J combination event, the same heavy chain complementary determining region (HCDR3) length, and HCDR3 amino acid sequences of 80% identity between different people (Figure 1(C)). We identified a notable clonotype, namely, VH3-53-J6, which exhibited stereotyped features of IGHV3-53, IGHJ6 (J6), and an 11 amino acid HCDR3, in 9 out of the 13 COVID-19 patient’s repertoires (Figure 1(D,E)). The VH3-53-J6 clonotype could be detected at ten days post symptom onset and continued to undergo clonal expansion during the disease course (Fig. S1A, Figure 1(F)). This VH3-53-J6 clonotype was present in 3 of 4 COVID-19 patient’s repertoires at three months after hospital discharge (Figure 1(F,G)), suggesting that the VH3-53-J6 clonotype persisted in the peripheral blood circulation for 3 months or longer. In contrast, this clonotype was not detectable in the repertoires of all five healthy donors (Fig. S1A, Figure 1(F)). These observations suggest that the VH3-53-J6 derived antibodies is preferentially induced in many people by SARS-CoV-2 infection.

We compared the VH3-53-J6 clonotype with the published RBD-targeted mAbs [3–15]. We found that many RBD-targeted mAbs (BD494, BD-500, BD-501, BD-503, BD-505, BD-506, BD507, BD508, CC12.1, C140, C210, COV2-2080, COV2-2037, COV2-2952, COV2-2165, and 1-20) were also derived from VH3-53/J6- combination, which has similar or identical HCDR3s as the VH3-53-J6 clonotype (Fig. S1B). Therefore, the VH3-53-J6 clonotype represents a highly shared antibody response to RBD in many SARS-CoV-2-exposed individuals. We next applied the known RBD-targeted VH3-53 mAbs [3–15] as references to query our repertoires. Several other VH3-53 clonotypes were found in the repertoires, which are highly similar to the known RBD-targeted mAbs COVA2-04, COVA2-13, BD-236, CV30 (Fig. S1C), B-38, COVA2-07 C148 (Fig. S1D), CC12.2, CC12.3, C003, C101, C102, C155, and C211 (Fig. S1E). Overall, pan-VH3-53 antibodies were highly prevalent among COVID-19 patients (Figure 1(E), Fig. S1B-1E) indicating a strong selection of VH3-53-encoded antibody response against the RBD of SARS-CoV-2.

### The VH3-53-J6 clonotype is derived from convergent rearrangements and exhibits evolutionary conservation

**Figure 2.**
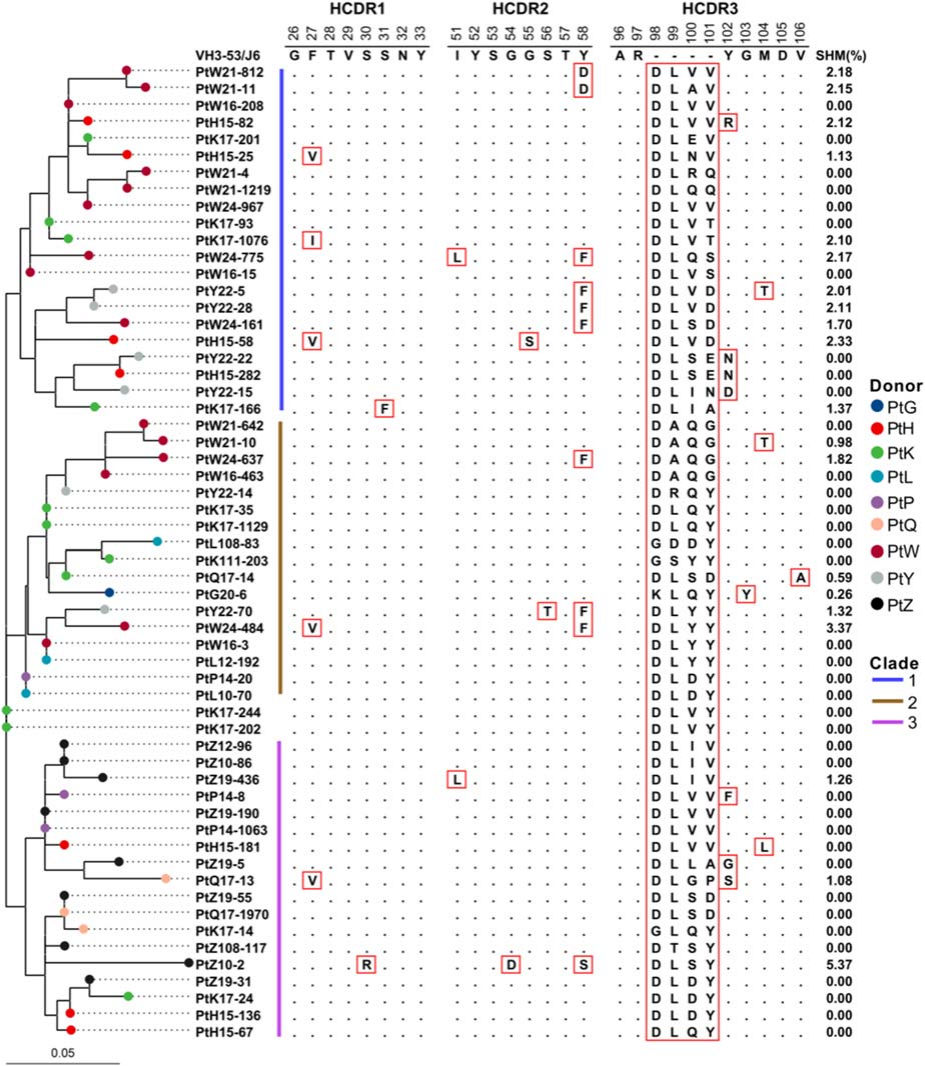
Sequence analysis of the VH3-53-J6 clonotype. Maximum-likelihood (ML) tree (left panel) and multiple alignments of amino acid sequences (middle panel) are shown. Somatic hypermutation rates of each sequence are also shown (right panel). Different coloured circles in the ML tree represent sequences that derived from different COVID-19 patients. Different coloured lines represent sequences that belong to different clades.

To better understand the convergence of VH3-53 antibodies in different donors, we constructed a cross-donor phylogenetic tree for the VH3-53-J6 clonotype (Figure 2). The maximum-likelihood (ML) method was used to reconstruct the geological tree. The result showed that sequences from different donors can be grouped into three major clades (Figure 2). These sequences were clustered according to lineage distribution rather than by individuals. We then compared the similarity of full-length amino acid sequence among the VH3-53-J6 clonotype. The similarity matrix showed that the VH3-53-J6 sequences from different people’s repertoires were very similar or even identical, with identity ranging from 88.9% to 100.0% (Fig. S2). Further analysis reveals that the convergence in HCDR1 and HCDR2 were higher than that in HCDR3 (Figure 2). The sequences on HCDR1 and HCDR2 regions were nearly identical to VH3-53 germline sequence (Figure 2). Most of the VH3-53-J6 sequences have no or limited somatic hypermutations (SHMs) in the variable region, ranging from 0.00% to 5.37% (Figure 2). This result indicates that these VH3-53 antibodies were likely derived from naïve B cells.

**Figure 3.**
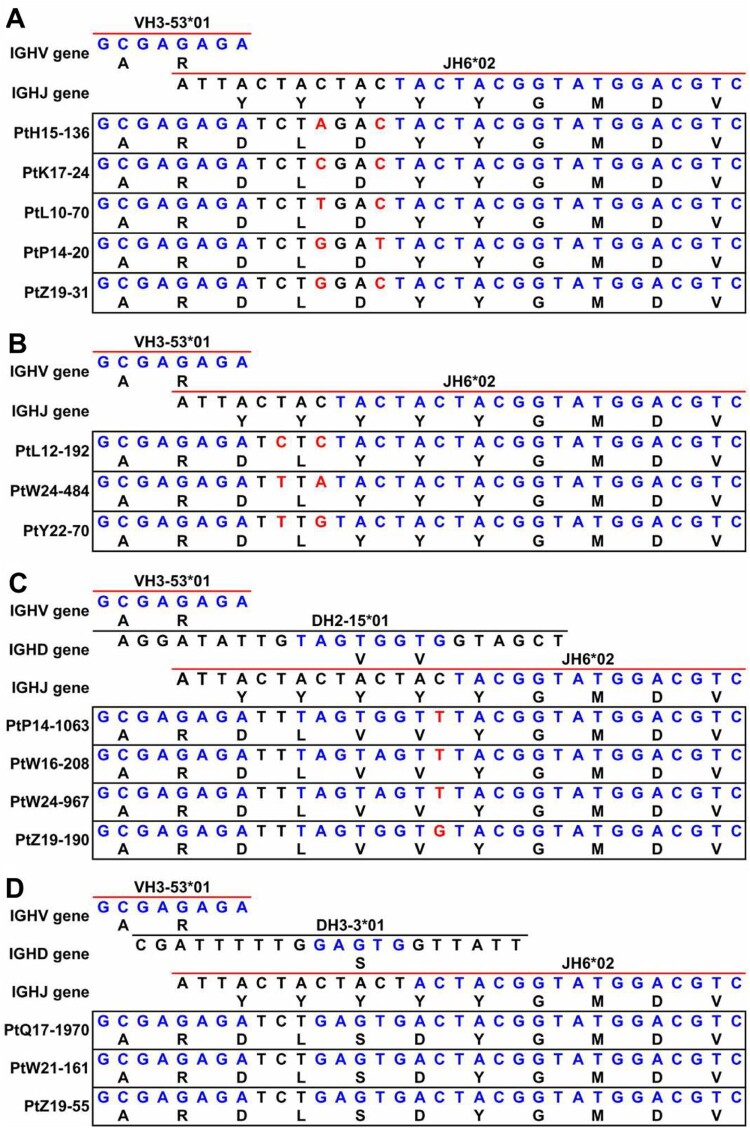
Scheme of germline gene rearrangements. Inferred germline V(D)J genes of closely related the VH3-53-J6 clonotype identified from different patient’s repertoires. Each block is labelled with the inferred germline sequences. Nucleotide positions and corresponding amino acid positions are labelled in blue colour if the sequences are same as the germline genes (top of each block). Nucleotide positions and corresponding amino acid positions are not coloured if they are inferred to be derived from un-templated nucleotide addition during V(D)J combination. Nucleotide positions are coloured with red colour if one amino acid is encoded by different synonymous codons. (A) Comparison of V(D)J gene rearrangement of PtH15-136, PtK17-24, PtL10-70, PtP14-20, and PtZ19-31. (B) Comparison of V(D)J gene rearrangement of PtL12-192, PtW24-484, and PtY22-70. (C) Comparison of V(D)J gene rearrangement of PtP14-1063, PtW16-208, PtW24-967, and PtZ19-190. (D) Comparison of V(D)J gene rearrangement of PtQ17-1970, PtW21-161, and PtZ19-55.

To further investigate the mechanism of the convergence of the VH3-53-J6 clonotype on HCDR3, we reconstructed the V/D/J germline rearrangement events. Two major rearrangement patterns were identified that lead to the constrained HCDR3 amino acid sequences. One rearrangement pattern contained VH3-53, J6 and a non-template sequence (Figure 3(A,B)). The other rearrangement pattern contained VH3-53, J6 and an inferred D gene segment (Figure 3(C,D)). Both combinations utilize translation frame 3 of J6 gene segment in their HCDR3s. This translation frame usually contains a common AR motif at the 5’ end and a common YGMDV motif at the 3’ end. The difference in these HCDR3s appears to result from the random insertion of non-template sequences or D gene segments (Figure 3(A–D)). In addition, synonymous codons were found to encode the same amino acid in the HCDR3 (Figure 3(A–C)). Taken together, these observations suggested the prevalent VH3-53-J6 clonotype among COVID-19 patients were generated by convergent evolution.

### Analysis of VH3-53 paired light chains reveals a shared VK1-9-J4 light-chain clonotype in the repertoires

**Figure 4.**
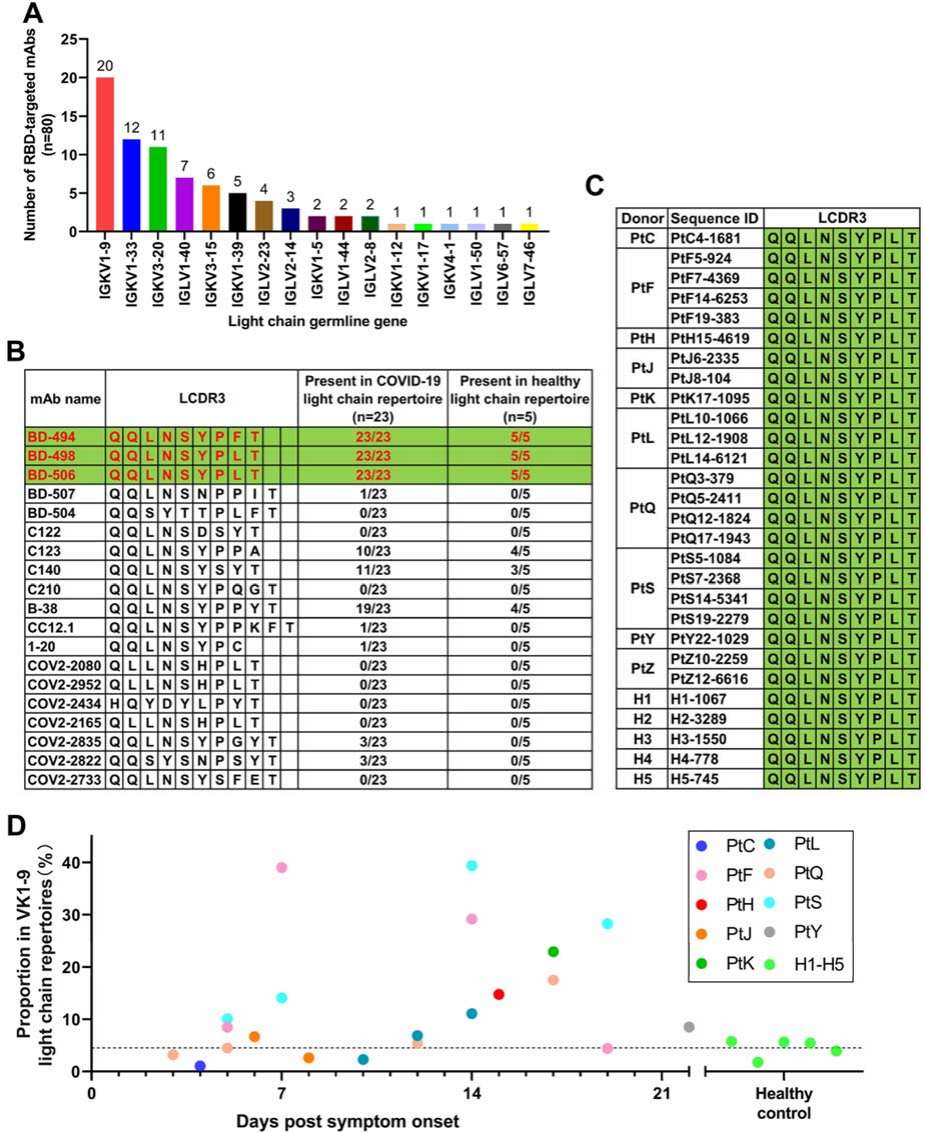
Light-chain VK1-9-encoded antibodies were highly shared among COVID-19 patients. (A) The usage distribution of light-chain germline gene that paired with VH3-53 in 80 known RBD-targeted antibodies[3, 4, 5, 6, 7, 8, 9, 10, 11, 12, 13, 14]. (B) Comparison of LCDR3 sequences of the known VK1-9-encoded light chains and their presence in the repertoires of COVID-19 patients and healthy people. (C) Comparison of LCDR3 sequences of the shared VK1-9-J4 clonotype identified from the repertoires of COVID-19 patients and healthy people. (D) The proportion of the VK1-9-J4 clonotype in the total VK1-9 light-chain repertoires of COVID-19 patients and healthy people.

We next analysed the germline gene usage of the light chains paired with VH3-53 encoded RBD-binding antibodies. Based on analysis of 80 RBD-targeted VH3-53 mAbs isolated from single B cells in 13 publications [3–15], we found that 17 different light-chain germline genes were used to pair with VH3-53 during natural pairings (Figure 4(A)). Among these light-chain germline genes, IGKV1-9 (VK1-9) is the most frequently observed. Using the known VK1-9-encoded light chains as references (Figure 4(B)), we identified a most shared VK1-9 light-chain clonotype, namely VK1-9-J4, which were present in all repertoires (Figure 4(C)). This clonotype was derived from the combination of VK1-9 and IGKJ4 (J4), which has an identical light-chain complementary determining region (LCDR3) as the published VH3-53 mAbs BD-498, BD-506 and only one amino acid mismatch with the LCDR3 of mAb BD-494 (Figure 4(B,C)). The VK1-9-J4 clonotype is constitutively expressed in every repertoire, but the abundance differs between COVID-19 and healthy samples (Figure 4(B–D)). Clonal expansion of the VK1-9-J4 clonotype was observed during SARS-CoV-2 infection (Figure 4(D)). Therefore, we synthesized the VK1-9-J4 light-chain sequence for pairing with repertoire-deduced VH3-53-J6 heavy chain sequences for functional characterization.

### The repertoire-deduced VH3-53-J6 heavy chains pairing with the common VK1-9-J4 light chain have RBD binding and neutralizing activities

**Figure 5.**
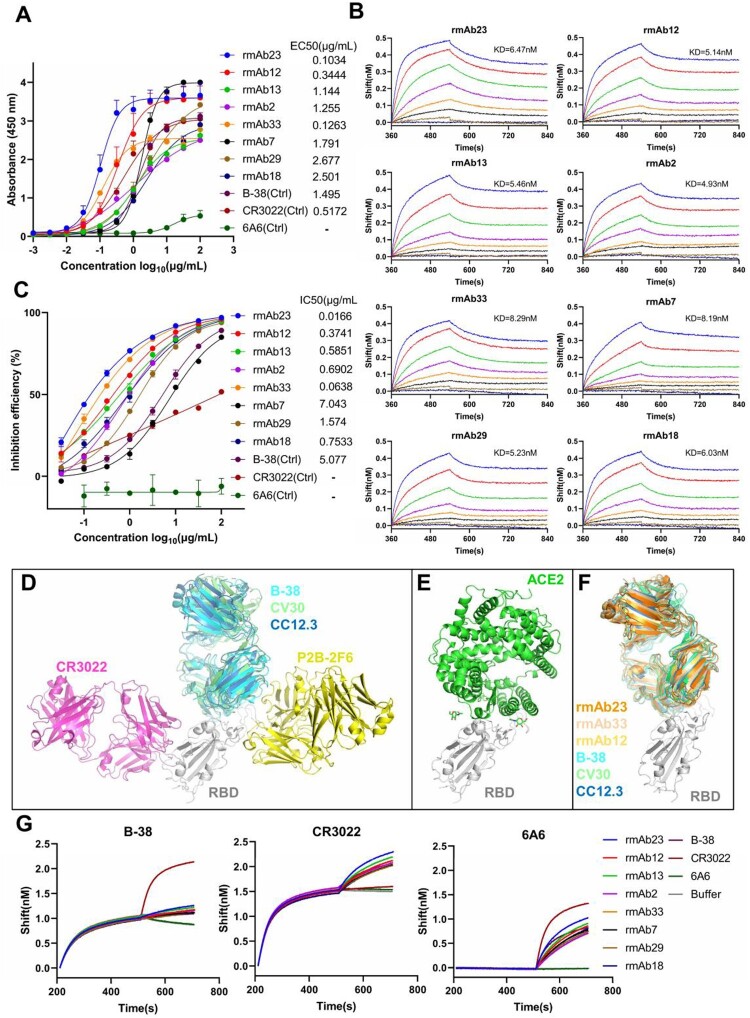
VH3-53-J6 antibodies identified from the repertoires of different COVID-19 patients can bind to SARS-CoV-2 RBD and block the interaction between RBD and ACE2. (A) Eight recombinant VH3-53 antibodies with relatively higher RBD-binding activities were selected for further analysis. Previously published mAbs B-38 [11], CR3022 [33] and 6A6 [47] were used as controls. (B) Binding kinetics of the 8 recombinant VH3-53 antibodies with SARS-CoV-2 RBD were measured by BLI. Immobilized individual antibody (10 μg/mL) was saturated with RBD at seven different concentrations (200 nM, 100 nM, 50 nM, 25 nM, 12.5 nM, 6.25 nM, and 3.125 nM) or buffer. (C) Neutralizing activities of the 8 recombinant VH3-53 antibodies, determined using a SARS-CoV-2 surrogate virus neutralization test (sVNT). B-38, CR3022 and 6A6 were used as controls (D) Comparison of B-38 (PDB: 7BZ5), CV30 (PDB: 6XE1) [17], CC12.3 (PDB: 6XC4) [16], CR3022 (PDB: 6W41), and P2B-2F6 (PDB: 7BWJ) [3] binding epitopes. SARS-CoV-2 RBD is shown in cartoon representation (grey). The heavy and light chains of B-38, CV30, CC12.3, CR3022, and P2B-2F6 are coloured in cyan, limegreen, skyblue, magenta, and yellow, respectively. (E) Crystal structure of ACE2 (green) in complex with SARS-CoV-2 RBD (grey) (PDB: 6LZG) [1]. (F) Comparison of rmAb23, rmAb33, rmAb12, B-38, CV30, and CC12.3 binding epitopes. Modelling structure of rmAb23, rmAb33, and rmAb12 in complex with SARS-CoV-2 RBD are coloured in orange, wheat, yellow-orange and grey, respectively. (G) Competition binding to RBD between the 8 recombinant VH3-53 antibodies and B-38, CR3022, or 6A6 was measured by BLI, respectively. Immobilized RBD (2 μg/mL) was saturated with 10 μg/mL of the first antibody and then flowed with equal concentration of the first antibody in the presence of or without the second antibody. The graphs show binding patterns after saturation of RBD.

To test whether the VH3-53-J6 clonotype deduced from the repertoires are reactive to RBD, we selected a subset of novel sequences for functional evaluation based on three selection criteria. First, VH3-53-J6 sequences with reads lower than 10 were excluded, since these sequences may not undergo clonal expansion after viral stimulation. Second, identical sequences detected at multiple time points were included since B cells expressing these sequences survived after antigen selection. Last, identical sequences present in multiple donors’ repertoires were included. Therefore, a total of 34 novel and shared heavy chain sequences were selected from multiple repertoires (Fig. S3A). We synthesized these novel VH3-53-J6 heavy chains and co-expressed with the shared VK1-9-J4 light chain in HEK293 cells to produce recombinant mAbs for functional analysis. Antibody binding activity to RBD was measured by enzyme-linked immunosorbent assay (ELISA) (Fig. S3B). Overall, 23 out of 34 recombinant mAbs showed binding activities to RBD, with rmAb23 (from patient W) having EC50 as low as at 0.1034 μg/mL (Fig. S3A-3B). Interestingly, several germline VH3-53-J6 heavy chains (PtK17-244, PtP14-1063, and PtW24-967, from 3 different patients) have no somatic hypermutations, even they bound to RBD with EC50 < 0.35 μg/mL (Figure 2, Fig. S5A). We next selected eight recombinant mAbs with relatively higher RBD-binding activities to test their affinity to SARS-CoV-2 RBD (Figure 5(A)). The eight recombinant mAbs showed high affinity to RBD with dissociation constants (KD values) ranging from 4.93 nM to 8.29 nM (Figure 5(B)). Neutralizing activities of the eight recombinant mAbs were determined using a SARS-CoV-2 surrogate virus neutralization test (sVNT), which was based on the blockage of ACE2 and RBD interaction [22]. All 8 RBD-binding recombinant mAbs showed good neutralizing activities in sVNT, with rmAb23 having IC50 at 0.0166 μg/mL, more potent than the previously published mAb B38 (5.077 μg /mL) (Figure 5(C)). We also verified the neutralizing activities of these 8 recombinant mAbs using a pseudovirus neutralization assay (Fig. S3C).

To identify potential epitopes recognized by these repertoire-deduced VH3-53 rmAbs, we take advantage of recently determined molecular interactions between the RBD and three VH3-53/J6-derived mAbs, B-38, CV30, and CC12.1 [10,16,17]. Superimposing the complex structures by aligning the RBDs reveals that B-38, CV30, and CC12.3 bind to the ACE2 binding site on RBD with in a similar manner (Figure 5(D,E)). However, two non-VH3-53-derived RBD-targeted mAbs, CR3022 [33] and P2B-2F6 [3], target to different epitopes as compared to B-38, CV30 and CC12.1 (Figure 5(D). We predicted the conformation of three top neutralizing mAbs, rmAb23, rmAb33, and rmAb12 in complex with RBD. The modeling structures suggested that rmAb23, rmAb33, and rmAb12 interact with RBD in a similar manner as previously cloned mAbs B-38, CV30, and CC12.3 (Figure 5(F)). We finally performed a competitive binding assay by biolayer interferometry (BLI) on the 8 RBD-binding mAbs against two known RBD-directed mAbs, B-38 and CR3022 [11,33]. All 8 RBD-binding VH3-53 mAbs could compete with B-38 but not with CR3022 (Figure 5(G)). Therefore, these repertoire-deduced mAbs could recognize the same epitope as B-38. Taken together, these results suggest that antibodies derived from VH3-53/J6 target the ACE2 binding site on RBD and can block the interaction between RBD and ACE2.

## Discussion

Based on computational analysis of 40 antibody repertoires from 13 patients, we identified common SARS-CoV-2 RBD-specific antibody repertoire signatures encoded by VH3-53 germline gene. A highly shared clonotype VH3-53-J6 (VH3-53, J6, and an 11 amino acid CDR3) was identified in 9 out of 13 SARS-CoV-2 exposed individuals, accounting for over 2/3 of COVID-19 patients. This shared VH3-53-J6 clonotype was derived from convergent gene rearrangements and was evolutionarily conserved. These VH3-53-J6 antibodies have no or few somatic hypermutations, suggesting that many people may have preprogramed in their germline sequence for rapid generation of RBD-targeted antibodies. Importantly, we were able to identify antigen-specific mAbs from millions of antibody sequences by mining antibody repertoires of COVID-19 patients. The repertoire-deduced VH3-53 heavy chain sequences that we identified can bind to RBD and have neutralizing activities, validating our finding of RBD-specific antibodies via repertoire sequencing and bioinformatic analysis.

This study highlights the prospect that antibody repertoire sequencing and computational analysis could identify RBD-specific mAbs among COVID-19 patients. Compared with the conventional antibody cloning technology, more than one million antibody sequences can be obtained in a parallel antibody repertoire sequencing, making in-depth studies of the repertoire possible. In addition, it allows to track the certain antibody lineages, such as the VH3-53-encoded antibodies. Although bulk sequencing loss, the information of paired heavy chain and light chain, it could be combined with single-cell 5′V(D)J sequencing or traditional single-cell RT-PCR technology.

Convergent antibody rearrangements were also observed in acute human dengue infection and human responses to influenza vaccination. Convergent antibody HCDR3 sequence (ARLDY_5_GMDL) is highly enriched in acute dengue samples [34]. While an overrepresentation of IGH, involving IGHV3-7, IGHJ6, and an 18 amino acid HCDR3, were observed in H1N1 responses [35]. These stereotypic antibody responses to infections provide evidence of convergent evolution, which are commonly elicited during infections. Reproducibly eliciting the particular antibody responses in different individuals during vaccination could be possible, since the immunoglobulin repertoire is generally large enough and the unexpectedly high prevalence of shared clonotypes are observed in human naïve B cell repertoires [36,37].

An important finding of this study is that the highly shared VH3-53 antibodies elicited in different COVID-19 patients target to the same epitope on SARS-CoV-2 RBD. The VH3-53 antibodies we identified from the repertoires have neutralizing activities through direct blocking of the hACE2–RBD interaction. These VH3-53 antibodies recognize the same epitope region as previously reported RBD-binding mAbs, B-38, CV30, and CC12.3. Antibodies that share the same germline gene usage and similar molecular interactions with antigens have been observed in other viral infections, including Ebola, Zika, and influenza, as well as chronic HIV-1 infection. The VH3-15/VL1-40-based class of antibodies targeting the NPC1-receptor-binding site on Ebola GP has been reported as a common response in individuals infected with the Ebola virus or vaccinated with Ebola virus vaccine rVSV-ZEBOV [38,39]. The VH3-23/VK1-5 antibodies specifically recognize the lateral ridge of ZIKV EDIII [40]. Influenza HA stem-directed broadly neutralizing antibodies frequently utilize certain germline genes, including VH1-69, VH1-18, VH3-30, and VH6-1 [41,42]. Broadly neutralizing antibodies targeting the CD4-binding site of HIV-1 are typically derived from VH1-2 or VH1-46 [43,44]. A common feature of these preferred germlines used for targeting viral glycoproteins at specific sites is that the interactions involve germline-encoded residues, which largely explain why certain germline genes are commonly used to target certain antigens. Notably, in both Ebola and Zika antibodies, the VH gene can only be paired with certain VL genes [8,38]. However, in both influenza and HIV-1 antibodies, the VH genes can be paired with different light chains. Structural analysis showed that the essential paratopes are mainly mediated by heavy chains in influenza and HIV-1 antibodies [44,45]. Like influenza and HIV-1 antibodies, the manner of interactions between the VH3-53-encoded mAbs and RBD are mainly determined by heavy chain [16]. Although VH3-53 is most frequently observed to pair with VK1-9, VH3-53 can also accommodate other light chains, which increases the probability of generating RBD-targeted antibodies. Our study showed that VH3-53-encoded neutralizing antibodies are broadly induced among COVID-19 patients, implying that the shared VH3-53 antibody response to SARS-CoV-2 may be a promising target for rational vaccine design.

Another important finding from the repertoire analysis is that the VH3-53 antibodies have very limited or even no somatic mutations. So far, most reported SARS-CoV-2-specific mAbs have no or few somatic hypermutations [5,10,12]. Notably, these SARS-CoV-2-reactive mAbs were isolated from the relatively early time points after infection, which may accumulate additional somatic mutations during affinity maturation later. However, through longitudinal tracking of 4 individuals for 3 months after hospital discharge, we did not observe additional somatic mutations on these VH3-53 antibodies in the repertoires (Figures 1(G) and 2). One limitation of this analysis is that only 4 patients were followed up to 3 months. The evolution of VH3-53-encoded in COVID-19 patients requires more systematic and in-depth research. In line with our findings, a recent report found that the VH3-53-encoded antibodies isolated at 6.2 months after infection also carry no or very limited somatic mutations in the paratope regions [46]. Importantly, we found that many unmutated VH3-53 antibodies can bind strongly to the RBD. The paratope residues used by VH3-53 antibodies for targeting SARS-CoV-2 RBD are already present in the germline sequence, further supporting that VH3-53 antibodies could develop rapidly and in high probability among many individuals [16]. This highlights the VH3-53 encoded antibodies as a critical mechanism of early response to SARS-CoV-2 infection. Germline-like potent neutralizing antibodies have also been described [20,47,48] in people infected with EBOV, ZIKV, and MERS-CoV. Our study suggested that there is a rapid extrafollicular immune response against SARS-CoV-2. The human B cell repertoires could mount “economic” germline gene against emerging SARS-CoV-2. Therefore, we could expect a fast-acting COVID-19 vaccine that can be designed to elicit neutralizing antibody response against SARS-CoV-2 by activating VH3-53 expression in naïve B cells without requiring extensive somatic mutations. Indeed, a recent study reported that the VH3-53-encoded antibodies account for about 10% of 1,409 mAbs isolated from the vaccinated individuals [49].

With the ongoing global spread of SARS-CoV-2, several viral variants emerged worldwide. Specifically, the B.1.1.7, B.1.351, and P.1 lineages that originated in the UK, South Africa, and Brazil, respectively, are characterized by the accumulation of mutations in spike protein, especially in the RBD. Three mutation K417N, E484K and N501Y in RBD of spike raised the most of concern [50–52]. Notably, several studies showed that VH3-53-encoded mAbs are generally resisted by mutations K417N and N501Y [53–55], suggesting that this class of antibodies induced in broad populations has a selective pressure on SARS-CoV-2. Future studies should address if SARS-CoV-2 variants can generate VH-3-53 antibodies with neutralizing potency. An ideal vaccine should be designed to elicit broad neutralizing antibodies that derived from a wide range of VH gene families.

Taken together, we revealed a convergent antibody response encoded by VH3-53-J6 clonotype to SARS-CoV-2. The VH3-53 antibodies provide a benchmark for evaluating SARS-CoV-2 and vaccine-induced antibody responses. Furthermore, our study demonstrated that antigen-specific mAbs can be digitally obtained by identifying shared clonotypes through antibody repertoire sequencing and computational analysis.

## Supplementary Material

Table_S1_editable.xlsx

## Data Availability

All materials that supported this study are available from the corresponding authors upon reasonable request. All raw antibody repertoire data used in this study have been deposited at the National Genomics Data Center (https://bigd.big.ac.cn/) under the accession number: PRJCA003775.
